# Time-Varying Vocal Folds Vibration Detection Using a 24 GHz Portable Auditory Radar

**DOI:** 10.3390/s16081181

**Published:** 2016-07-28

**Authors:** Hong Hong, Heng Zhao, Zhengyu Peng, Hui Li, Chen Gu, Changzhi Li, Xiaohua Zhu

**Affiliations:** 1 School of Electronic and Optical Engineering, Nanjing University of Science and Technology, Nanjing 210094, China; hongnju@njust.edu.cn (H.H.); soniczhao@live.com (H.Z.); lihui_njust@126.com (H.L.); gc_njust@163.com (C.G.); zxh_njust@126.com (X.Z.); 2 Department of Electrical and Computer Engineering, Texas Tech University, Lubbock, TX 79409, USA; zhengyu.peng@ttu.edu

**Keywords:** non-acoustic, vocal folds, fundamental frequency, VMD, auditory radar

## Abstract

Time-varying vocal folds vibration information is of crucial importance in speech processing, and the traditional devices to acquire speech signals are easily smeared by the high background noise and voice interference. In this paper, we present a non-acoustic way to capture the human vocal folds vibration using a 24-GHz portable auditory radar. Since the vocal folds vibration only reaches several millimeters, the high operating frequency and the 4 × 4 array antennas are applied to achieve the high sensitivity. The Variational Mode Decomposition (VMD) based algorithm is proposed to decompose the radar-detected auditory signal into a sequence of intrinsic modes firstly, and then, extract the time-varying vocal folds vibration frequency from the corresponding mode. Feasibility demonstration, evaluation, and comparison are conducted with tonal and non-tonal languages, and the low relative errors show a high consistency between the radar-detected auditory time-varying vocal folds vibration and acoustic fundamental frequency, except that the auditory radar significantly improves the frequency-resolving power.

## 1. Introduction

Speech, human’s most used means of communication, has been the object of intense study for more than 150 years. Much significant progress has been made in speech signal processing, such as speech synthesis, speech recognition, speech enhancement, speech coding, speaker identification, etc. [1]. As we know, the microphone is the most common device to record the speech signals. However, the recorded speech signals are easily smeared by the high background noise and voice interference, which will considerably degrade the quality of the recorded signals. Since the speech signal and noise always have the same frequency band, it becomes very difficult to separate speech signals from high background noise, which gains more and more attention [2,3].

In the past two decades, studies using non-acoustic sensors have shown that the glottal excitation and vocal folds articulator movements can be measured in real-time as an acoustic speech signal is produced [4]. Relevant non-acoustic sensors could be classified into two categories: the physical instruments and microwave devices. In physiology, instruments including the electroglottography (EGG) [5,6], throat microphones [7], and bone-conduction microphones [8,9] have been proposed to detect the motion of human vocal folds. From the microwave devices, the general electromagnetic motion sensor (GEMS) attracts considerable concern [6,8,10], which can be used to measure tissue movements during voiced speech and speech involving vocal folds vibration. With GEMS, an antenna is typically strapped on the throat at the laryngeal notch or other facial locations. However, most of the physical instruments and GEMS need to be placed on the skin or close to the mouth, which makes the user discomfort and leads to the skin irritation.

In recent years, biomedical radar technology has attracted great interest in various fields, such as medical monitoring, military applications, etc. [11,12,13,14,15,16,17]. In medical monitoring, this technique could not only free the subject from being planted with directly contacted sensors, but also widen the monitoring period and avoid the measurement bias because of psychological stress. In military applications, such a technique can be used to find hidden enemies behind walls, or rapidly evaluate the status of victims on the battlefield.

More recently, biomedical radar technology has been extended to detect the speech signal information. In [18], a novel millimeter microwave radar was proposed to detect speech signals. The acquired speech quality is comparable to the microphone signal. Incorporating with this radar, this group used a higher-order statistics algorithm to enhance the speech quality [19]. Moreover, they presented a 94-GHz radar in [20] and an algorithm to improve the detection quality [21]. The results show that the noise is greatly suppressed. However, these works focus on the suppression of background noise. In [22], a 925-MHz speech radar system was proposed. From the system, the speech induced vocal vibration can be detected reliably. However, the experiments just reveal the similarity of radar-detected and microphone-detected signals. In our previous work, we showed the feasibility of detecting human speech via the biomedical radar and demonstrated its advantages of noise rejection and directional discrimination [23]. However, the inherent relationship between radar-detected and microphone-detected signals needs to be further explored before it can be used widely.

Therefore, all these limitations necessitate a reliable radar system that can capture the tiny human vocal folds vibration, and an accurate signal processing algorithm that can realize high frequency resolving power to demonstrate the inherent time-varying characteristics of radar-detected signal. In this paper, a 24-GHz portable auditory radar system is designed and fabricated. The high operating frequency and the 4 × 4 array antennas are applied to achieve the high sensitivity. The Variational Mode Decomposition (VMD) based algorithm is proposed to decompose the radar-detected auditory signal into a sequence of intrinsic modes firstly, and then, extract the time-varying vocal folds vibration frequency from the vocal folds vibration bearing mode. The VMD algorithm is entirely non-recursive and shows attractive performance with respect to existing decomposition models [24,25]. Its model provides a solution to the decomposition problem that is theoretically well founded. The basic detection theory and the radar hardware are presented in Section 2. The VMD based algorithm is described in Section 3. Then, in Section 4, two sets of experiments on non-tonal language (English) and tonal language (Chinese) are presented. Finally, a conclusion is drawn in Section 5.

## 2. Auditory Radar Theory

### 2.1. Basic Detection Theory

**Figure 1:**
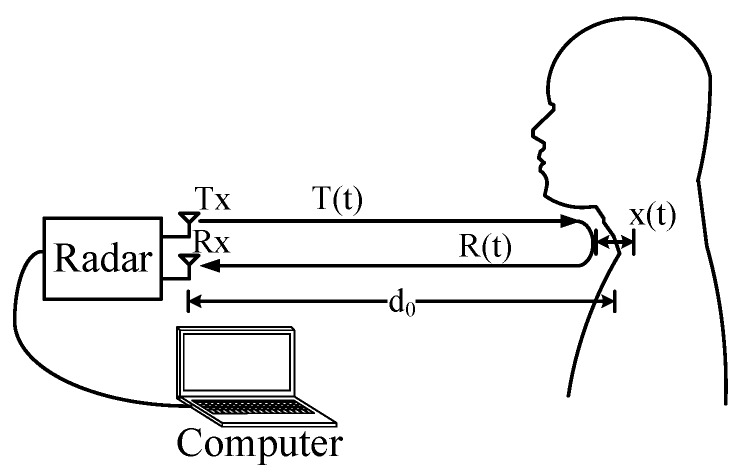
The basic mechanism of the auditory radar.

The auditory radar is functioning based on the phase estimation of the signals reflected by the vibrating vocal folds. The block diagram of the auditory radar system is illustrated in Figure 1.

The auditory radar typically transmits a radio-frequency continuous wave (CW) signal as follows:

(1) T(t)=cos[2πft+Φ(t)]

where *f* is the carrier frequency and Φ(t) is the phase noise. If the human subject is located $d_{0}$ away from the radar with the vocal folds vibration x(t), the total transmitted distance becomes $2d\left(t\right)=2d_{0}+2x\left(t\right)$. Thus, the reflected signal captured by the radar sensor at the moment *t* is actually the signal transmitted at the moment t-2d(t)/c, where *c* is the signal’s propagation speed (i.e., speed of light in free space). As a result, the reflected signal captured by the radar sensor at the moment *t* can be written as [26]:

(2) $$R\left(t\right)=T\left(t-\frac{2d(t)}{c}\right)=\cos\left[2\pi f\left(t-\frac{2d(t)}{c}\right)+\Phi \left(t-\frac{2d(t)}{c}\right)\right]$$

Substituting d(t) with $d_{0}+x\left(t\right)$, the received signal can be further written as:

(3) $$R\left(t\right)=\cos[2\pi ft-\frac{4\pi d_{0}}{\lambda }-\frac{4\pi x(t)}{\lambda }+\Phi \left(t-\frac{2d_{0}}{c}-\frac{2x(t)}{c}\right)]$$

where λ=c/f is the wavelength. Because the vocal folds vibration x(t) is much smaller than nominal detection distance of $d_{0}$, the change of the phase noise Φ is negligible. Therefore, the received signal can be finally approximated as:

(4) $$R\left(t\right)\approx \cos[2\pi ft-\frac{4\pi d_{0}}{\lambda }-\frac{4\pi x(t)}{\lambda }+\Phi \left(t-\frac{2d_{0}}{c}\right)]$$

The received radio frequency (RF) signal is down-converted to a baseband directly by mixing with the local oscillator (LO) signal T(t). In order to avoid the optimal/null point problem, the quadrature architecture is adopted in the radar [26]. As a result, the baseband quadrature signals can be written as [26]:

(5) $$B_{I}\left(t\right)=\cos\left[\theta +\frac{4\pi x(t)}{\lambda }+\Delta \Phi \left(t\right)\right]$$

(6) $$B_{Q}\left(t\right)=\sin\left[\theta +\frac{4\pi x(t)}{\lambda }+\Delta \Phi \left(t\right)\right]$$

where $\theta =4\pi d_{0}/\lambda +\theta_{0}$ is the constant phase shift depending on the nominal distance to the target $d_{0}$, $\Delta \Phi \left(t\right)=\Phi \left(t\right)-\Phi \left(t-2d_{0}/c\right)$ is the total residual phase noise.

As we can see, the vocal folds vibration is involved in the phase of baseband signals. In practice, when a human speaks, the vibration displacement x(t) is non-sinusoidal [22]. It is well-known that this non-sinusoidal waveform contains the fundamental frequency of the speech signal, which is variable when the words or the tone changes. To extract the phase information, the complex signal demodulation (CSD) method is used to combine the quadrature channel outputs as [27]:

(7) $$\begin{array}{cc} S(t) & =B_{I}\left(t\right)+j\cdot B_{Q}\left(t\right)\\ & =\cos\left[\theta +\frac{4\pi x(t)}{\lambda }+\Delta \Phi \left(t\right)\right]+j\cdot \sin\left[\theta +\frac{4\pi x(t)}{\lambda }+\Delta \Phi \left(t\right)\right]\\ & =\exp\{j\left[\frac{4\pi x(t)}{\lambda }+\Delta \Phi \left(t\right)\right]\} \end{array}$$

As demonstrated in [27], the CSD is immune from the direct current (DC) offset but can be affected by noise due to random body movement. The high operating frequency makes the radar very sensitive to random body movement.

### 2.2. The 24 GHz Portable Auditory Radar

**Figure 2:**
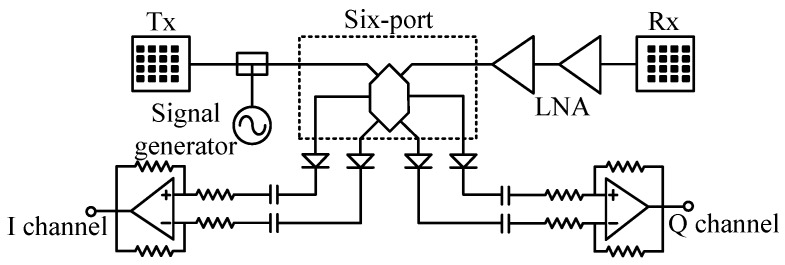
The block diagram of the 24-GHz auditory radar.

**Figure 3:**
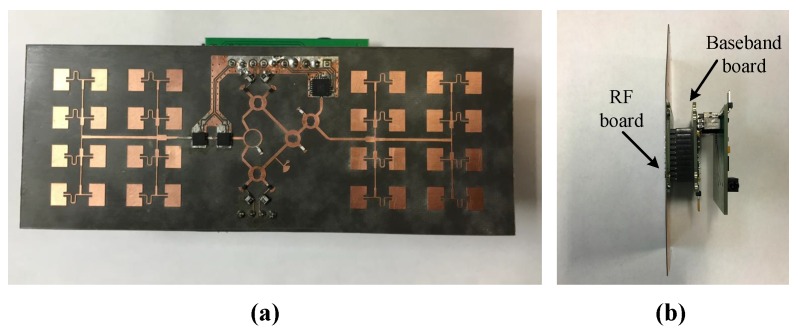
The photographs of the 24-GHz auditory radar from (**a**) front side and (**b**) right hand side.

Figure 2 and Figure 3 present the block diagram and photographs of the portable auditory radar, respectively. The transmit power is 8 dBm and the DC power consumption is 1.1 W. The carrier frequency used in this work is 24 GHz, which has a *μ*m-scale motion detection sensitivity [28]. The RF signal is divided into two channels, one is transmitted through the transmitting antenna and the other serves as the local oscillator (LO) signal in the receiver chain.

To enhance the directivity, a pair of 4 × 4 antenna arrays are designed, offering an antenna directivity of 19.8 dBi. As shown in Figure 3a, the antenna arrays are fabricated and integrated on a Rogers RT/duroid 5880 flexible microwave substrate (Chandler, AZ, USA) along with the RF front-end, which reduces the total device size to 11.9 cm × 4.4 cm.

In the receiver chain, the received signal is first amplified by two-stage low noise amplifiers (LNAs). Compared with the existing integrated mixer chips at 24 GHz, the six-port structure is simpler and cheaper. The outputs of the six-port downconverter are differential quadrature signals, which are amplified by two differential amplifiers to generate the baseband *I/Q* signals. The received RF gain and baseband gain are 34 dB and 26 dB, respectively. The baseband signals are fed to a 3.5 mm audio jack, which can be easily connected to the audio interface of a laptop or a smart phone for real-time signal processing.

## 3. Algorithm and Its Implementation

In this section, we start by merging the basic theory of Variational Mode Decomposition (VMD) into the time-varying vocal folds vibration detection framework [24]. Then, the details of the implementation are described. Finally, an example is presented for illustration.

### 3.1. VMD

The goal of the VMD is to decompose a real valued input signal *f* into *K* discrete number of modes $u_{k}$ (k=1,2,3,…,K), that have specific sparsity properties, so that the original signal can be reconstructed as:

(8) $$f\left(t\right)=\sum_{k}u_{k}$$

Each mode $u_{k}$ is assumed to be mostly compact around a center angular frequency $\omega_{k}$, which is determined along with the decomposition. The VMD algorithm to assess the bandwidth of a one dimension signal is as follows: (1) for each mode $u_{k}$, compute the associated analytic signal through Hilbert transform in order to obtain a unilateral frequency spectrum; (2) for each mode $u_{k}$, shift the mode’s frequency spectrum to baseband by means of mixing with an exponential tuned to the respective estimated center frequency; and (3) estimate the bandwidth through Gaussian smoothness of the demodulated signal, i.e., the squared $L^{2}$-norm of the gradient. Then, the constrained variational problem is given as follows:

(9) $$\min_{u_{k},\omega_{k}}\{\sum_{k}∥\partial_{t}\left[\left(\delta \left(t\right)+\frac{j}{\pi t}\right)*u_{k}\left(t\right)\right]e^{-j\omega_{k}t}{∥}_{2}^{2}\}$$

where $\left\{u_{k}\right\}=\left\{u_{1},\ldots ,u_{K}\right\}$ and $\left\{\omega_{k}\right\}=\left\{\omega_{1},\ldots ,\omega_{K}\right\}$ are shorthand notations for the set of all modes and their center frequency, *δ* is the Dirac distribution, *t* is time script, *k* is the number of modes, and * represents convolution.

In the VMD framework, the original real valued input signal *f* is decomposed into a set of *k* modes $u_{k}$ each having a bandwidth in Fourier domain and compacted around a center angular frequency $\omega_{k}$. The solution to the original minimization problem is the saddle point of the following augmented Lagrangian (*L*) expression:

(10) $$L\left(u_{k},\omega_{k},\lambda \right)=\alpha \sum_{k}∥\partial_{t}\left[\left(\delta \left(t\right)+\frac{j}{\pi t}\right)*u_{k}\left(t\right)\right]{∥}_{2}^{2}+{∥f-\sum u_{k}∥}_{2}^{2}+\langle \lambda ,f-\sum u_{k}\rangle$$

where *λ* is the Lagrange multiplier and ${∥•∥}_{p}$ denotes the usual vector $\ell_{p}$ norm, where p=2. The solution to Equation (8) are found in a sequence of *k* iterative sub-optimizations. Finally, the solutions for *u* and *ω* are found in Fourier domain and are given by:

(11) $$u_{n}^{n+1}=\left(f-\sum_{i\neq k}u_{i}+\frac{\lambda }{2}\right)\frac{1}{1+2\alpha {\left(\omega -\omega_{k}\right)}^{2}}$$

(12) $$\omega_{n}^{n+1}=\frac{\int_{0}^{\infty }\omega {\left|u_{k}\left(\omega \right)\right|}^{2}d\omega }{\int_{0}^{\infty }{\left|u_{k}\left(\omega \right)\right|}^{2}d\omega }$$

where *α* is known as the balancing parameter of the data-fidelity constraint, and *n* is the number of iterations. More details could be found in [24]. We therefore are provided with an opportunity to analyze the received signal R(t) from few individual modes that are remarkably simpler than the original R(t).

### 3.2. Implementation

**Figure 4:**
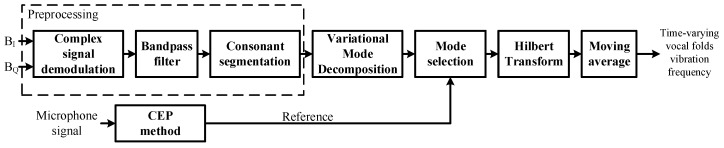
The flowchart of the signal processing algorithm.

The flowchart of the proposed signal processing algorithm is shown in Figure 4. The details of the implementation of the algorithm are described as follows.

#### 3.2.1. Preprocessing

The complex signal demodulation method is used to combine the quadrature channel outputs $B_{I}$ and $B_{Q}$. After complex signal demodulation, the demodulated signal S(t) is filtered by a bandpass filter (50–1500 Hz) to reduce the magnitude of both the DC component and the random body movement. Then, the filtered signal s(t) is preprocessed by consonant segmentation so as to remove those segments that are identified as being silent or unvoiced. Here, we adopt the short-term energy techniques to realize the segmentation [1]. In the meantime, applying the cepstrum (CEP) method to the microphone-detected signal yields a set of discrete values of the vocal folds vibration frequency [29,30,31]. Let $f_{v}$, (v=1,2,3,…,V) denote those of the non-vanishing values in the data set. Then, the mean value ${\bar{f}}_{v}$ is calculated as:

(13) $${\bar{f}}_{v}=V^{-1}\sum_{v}f_{v}$$

which is represented as the reference to locate the mode that contain the vocal folds vibration information.

#### 3.2.2. Decomposition and Mode Selection

The preprocessed radar signal r(t) is decomposed with the VMD as described above, yielding a set of modes, $u_{k}$ (k=1,2,3,…,K). In this study, the moderate bandwidth constraint *α* is set to 2000, the noise-tolerance *τ* is taken as 0, the number of modes *k* is arbitrarily set to 2, the center frequencies $\omega_{k}$ are uniformly initialized, the tolerance of the convergence criteria tol is taken as 1e-7 without imposing any DC component.

Then, there arises a crucial issue on how to single out, among the modes, the one or the ones that contain the time-varying vocal fold vibration information. We tackle this issue by catching the time-varying trend of vocal folds vibration and using it as a reference to track the variation of vocal folds vibration. This reference is evaluated by means of the CEP method, which was mentioned before. Finally, one of the decomposed modes whose average frequency is the closest to the reference ${\bar{f}}_{v}$ is picked up for the vocal folds vibration bearing mode, which can be described as:

(14) $$\underset{k}{\mathrm{argmin}}|\bar{f_{v}}-\max\left[F\left(u_{k}\right)\right]|$$

where F(·) denotes the Fourier transform.

#### 3.2.3. Calculation of Time-Varying Vocal Folds Vibration Frequency

The time-varying frequency, $\hat{f}\left(t\right)$, is calculated via Hilbert Transform [32]. To eliminate the possible random fluctuation induced by noise and computational errors, these time-varying frequency curves are smoothed by a simple averaging after windowing:

(15) $$f_{i}\left(t\right)=\frac{1}{T}\int_{-T/2}^{+T/2}\hat{f}\left(\tau \right)W\left(\tau -t\right)d\tau$$

where W(t) is a rectangular window function of width *T* and height 1, with *T* set to be three times of the dominant oscillatory period of the selected mode.

### 3.3. Illustration

**Figure 5:**
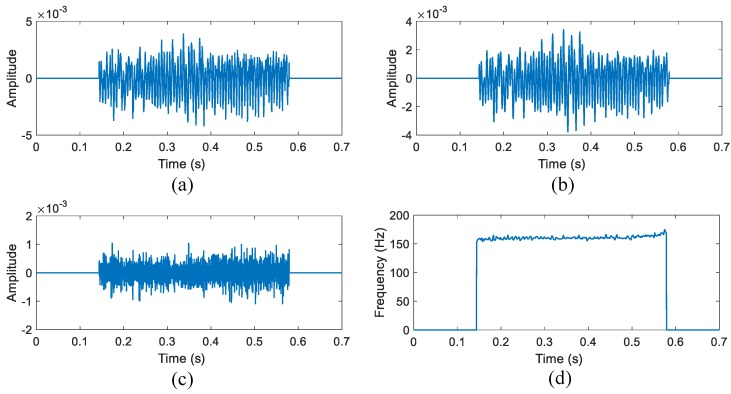
The results from the Variational Mode Decomposition (VMD)-based algorithm of a single character "A". (**a**) The phase information after segmentation; (**b**) The first mode decomposed by the VMD; (**c**) The second mode decomposed from the VMD; and (**d**) the time-varying frequency of the signal in (**a**).

An illustration of the operation is shown in Figure 5. Here, we take the decomposition of English character "A" as an example. Figure 5a shows the demodulated phase information after consonant segmentation of the English character "A", and Figure 5b,c display the two modes decomposed by means of VMD. Figure 5b shows that only the first mode contains the time-varying vocal folds vibration information, while Figure 5c is the noise. Then, the time-varying frequency is captured successfully as shown in Figure 5d.

## 4. Experiments

In this section, two sets of experiments are carried out. In the experiment, two healthy subjects (Subject A: male, 26-year-old; Subject B: male, 27-year-old) were asked to each sit on a chair and read the required characters, words or phrases. The auditory radar was placed 40 cm away from the human subject with its array antennas facing the subject’s throat. For comparison, a smart phone was placed 40 cm away to record the acoustic signal simultaneously.

### 4.1. Non-Tonal Language

English, which is known as a typical non-tonal language, was tested firstly. In the first set of experiments, the subject read some English characters, words and a sentence in standard speed and intonation. The experiments were conducted in a quiet room to reduce the acoustic interference. The radar-detected time-domain waveform after filtering and segmentation is shown in Figure 5a, along with the decomposed modes in Figure 5b,c. As we analyzed before, the vibration-related periodic oscillation only exists in the first mode, while the second mode is noise. The hand-labeled method is used to extract the microphone-detected fundamental frequency for comparison, which is known as one of the most accurate methods in speech signal processing [29]. First, we manually find out each peak or valley in the time-domain speech signal. Then, the discrete points are sorted as a series $t_{i}$ (i=1,2,3,…,N). As a result, the discrete fundamental frequency can be found as:

(16) $$f\left(t=\frac{t_{i}+t_{i+1}}{2}\right)=\frac{1}{▵t}=\frac{1}{t_{i+1}-t_{i}}$$

**Figure 6:**
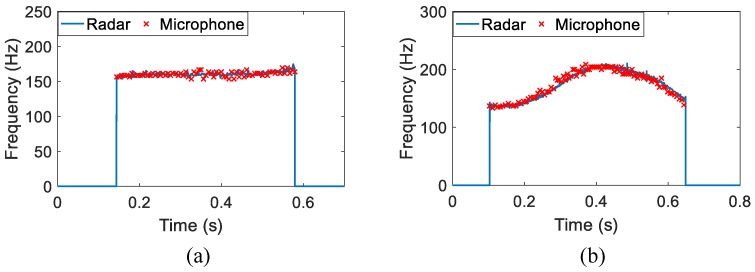
The auditory radar-detected time-varying vocal folds vibration frequency of one English character and one English word: (**a**) "A" and (**b**) "hello". The "×" symbols represent the hand-labeled acoustic fundamental frequency values.

The time-varying result of the spoken character "A" is shown in Figure 6a. Also plotted in the figures for comparison are those hand-labeled fundamental frequency values of microphone-detected speech signals. From this figure, we can see the radar-detected vocal folds vibration frequency is located around 170 Hz, and the trend of time-varying envelop fits the hand-labeled acoustic fundamental frequency values well, except that the auditory radar greatly improves the frequency-resolving power. Figure 6b presents the comparative result of a word "hello". Similarly, the radar-detected frequency closely matches the microphone-detected one. Moreover, a rising and a falling can be observed in the time-varying frequency. It indicates the frequency variations of the two different vowels in this word.

To further demonstrate the effectiveness of the auditory radar, intensive tests were performed for seven characters and two words. Here, we define the deviation degree of the hand-labeled fundamental frequency values as relative error:

(17) $$e=\frac{1}{N}\sum_{1}^{N}\frac{\left\{f_{r}\left(t=t_{n}\right)-f_{v}\left[n\right]\right\}}{f_{v}\left[n\right]}*100\%$$

where $f_{r}\left(t\right)$ means the time-varying vocal folds vibration frequency at the moment $t_{n}$, n=1,2,3......N. Table 1 summarizes the relative errors of seven characters and two words. From this table, we find that the relative errors of the character and word are below 10%. It is well-known that there are more consonants in a word than a single character. When the subject pronounces the consonants, the vocal folds vibration becomes disordered and non-vibratory, which may be smeared or difficult to capture by the radar. The low relative errors show a high consistency between the radar-detected vibration and acoustic fundamental frequency. In addition, the durations of these English characters and words are given in the table to illustrate the difference between characters and words.

**Figure 7:**
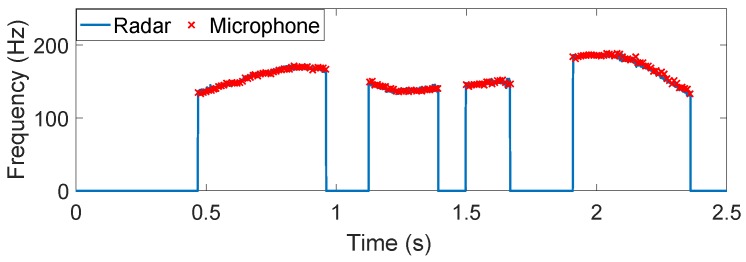
The auditory radar-detected time-varying vocal folds vibration frequency of the continuous speech signal, "I am a boy". The "×" symbols represent the hand-labeled acoustic fundamental frequency values.

Finally, we show a more general example of detecting the time-varying vocal folds vibration of an English sentence "I am a boy". The results are illustrated in Figure 7. After the segmentation, four separated segments can be found, which are related to the four words in the sentence. We can observe the intonation variation in each word. In addition, the time-varying vocal folds vibration captured by the presented method agrees very well with the hand-labeled acoustic fundamental frequency track, whether for English characters "I" and "a" or two words "am" and "boy".

### 4.2. Tonal Language

Reliable detection of the time-varying vocal folds vibration frequency is of crucial importance in speech processing of tonal languages as Chinese. In the second set of experiments, we tested some Mandarin Chinese monosyllable, multisyllabic word and continuous speech in standard speed and intonation. The experiments were also conducted in a quiet room to reduce the noise interference. We utilized the 24-GHz portable auditory radar to capture the vocal folds vibration of monosyllable and disyllable, and then applied the proposed method to signal processing to examine whether it could extract the time-varying vocal folds vibration information.

**Figure 8:**
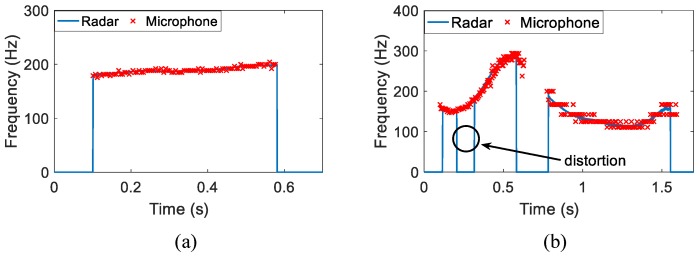
The auditory radar-detected time-varying vocal folds vibration frequency of one Chinese monosyllable and one Chinese disyllable: (**a**) /ti$\bar{a}$n/ and (**b**)/p$\overset{´}{i}$ng gu$\overset{˘}{o}$/. The "×" symbols represent the hand-labeled acoustic fundamental frequency values.

Figure 8 presents the time-varying vocal folds vibration frequency detected using the new scheme for one Chinese monosyllable and one Chinese disyllable: (a) /ti$\bar{a}$n/ in Chinese, meaning “Sky” in English and (b) /p$\overset{´}{i}$ng gu$\overset{˘}{o}$/ in Chinese, meaning “Apple” in English. Those hand-labeled fundamental frequency values of microphone-detected speech signals are plotted in the figures for comparison. For /ti$\bar{a}$n/, the time-varying vocal folds vibration frequency fits the hand-labeled acoustic fundamental frequency values very well as shown in Figure 8a, except that the auditory radar greatly improves the frequency-resolving power. For Chinese disyllable /p$\overset{´}{i}$ng gu$\overset{˘}{o}$/, the hand-labeled fundamental frequency values are distorted due to the limitation of significant digits; however, the auditory radar gives an encouraging result that correctly delineates the temporal variation of this disyllable. Note that the distortion in Figure 8b is due to the low sound intensity.

To further demonstrate the effectiveness of the auditory radar, intensive tests were performed for seven monosyllables, one disyllable and one trisyllable. Table 2 summarizes the relative errors of these syllables. From this table, it can be observed that the relative errors of the monosyllables and multisyllabic words are below 10%. The low relative errors show a high consistency between the radar-detected time-varying vocal folds vibration and acoustic fundamental frequency. In addition, the durations of these syllables are given in the table to illustrate the difference between monosyllable and multisyllabic words.

**Figure 9:**
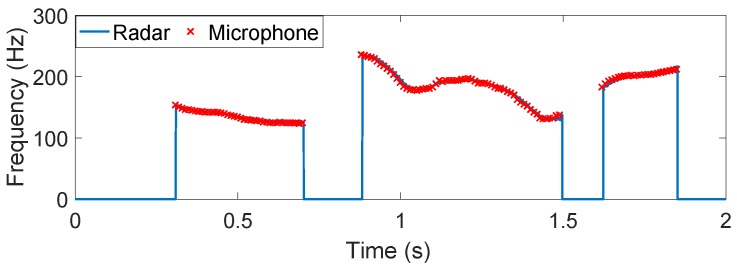
The auditory radar-detected time-varying vocal folds vibration frequency of the continuous speech signal, ’/q$\overset{ˇ}{i}$ng zh$\overset{`}{u}$ y$\overset{`}{i}$ $\bar{a}$n qu$\overset{´}{a}$n/’. The "×" symbols represent the hand-labeled acoustic fundamental frequency values.

Finally, we present a more general example of detecting the time-varying vocal folds vibration of a continuous Chinese speech signal, which is the sentence ’/$q\overset{ˇ}{i}\mathrm{ng}$ $\mathrm{zh}\overset{`}{u}$ $y\overset{`}{i}$ $\bar{a}n$ $\mathrm{qu}\overset{´}{a}n$/’ in Chinese, meaning ’Attention please’. As shown in Figure 9, the present method results in the dynamic structures of time-varying vocal folds vibration, which agrees very well with the hand-labeled acoustic fundamental frequency track, whether for single word /$q\overset{ˇ}{i}\mathrm{ng}$/ or two phrases /$\mathrm{zh}\overset{`}{u}$ $y\overset{`}{i}$/ and /$\bar{a}n$ $\mathrm{qu}\overset{´}{a}n$/.

## 5. Conclusions

This paper has presented a non-acoustic way to capture the human vocal folds vibration using a 24-GHz portable auditory radar. The auditory radar is highly integrated with a small size and low power consumption. The VMD based algorithm is proposed to decompose the radar-detected auditory signal into a sequence of intrinsic modes. Therefore, the time-varying vocal folds vibration frequency is extracted from the corresponding mode. The low relative errors show a high consistency between the radar-detected auditory time-varying vocal folds vibration and acoustic fundamental frequency, which enable potential applications in robust speech recognition, recovery and surveillance.

## Figures and Tables

**Table 1:** The relative errors of the tested English characters and words. ("A","B","C","D","F","hello" and "boy" are from Subject A. "E" and "O" are from Subject B.)

Character/Word	A	B	C	D	E	F	O	Hello	Boy
**Duration/s**	0.44	1.07	0.41	0.66	0.41	0.08	0.29	0.55	0.81
**Relative error**	3.23%	4.45%	1.47%	4.09%	6.22%	3.89%	2.49%	5.46%	9.88%

**Table 2:** The relative errors of Chinese monosyllables and multisyllabic words. (/n$\overset{˘}{i}$/, /x$\overset{´}{i}$ng/, /w$\overset{˘}{o}$/, /w$\overset{˘}{o}$/, /h$\overset{˘}{a}$o/, /t$\bar{i}$an/, /p$\overset{´}{i}$ng gu$\overset{ˇ}{o}$/ and /z$\overset{˘}{a}$o sh$\overset{`}{a}$ng h$\overset{ˇ}{a}$o/ are from Subject A. /h$\bar{a}$o/ and /y$\overset{˘}{o}$u/ are from Subject B.)

Character/Word	/n$\overset{˘}{i}$/	/x$\overset{´}{i}$ng/	/w$\overset{˘}{o}$/	/h$\overset{˘}{a}$o/	/h$\bar{a}$o/	/y$\overset{˘}{o}$u/	/t$\bar{i}$an/	/p$\overset{´}{i}$ng gu$\overset{ˇ}{o}$/	/z$\overset{˘}{a}$o sh$\overset{`}{a}$ng h$\overset{ˇ}{a}$o/
**Duration/s**	0.43	0.448	0.188	0.415	0.21	0.17	0.48	1.44	1.66
**Relative error**	1.62%	2.36%	1.96%	8.31%	2.29%	6.06%	1.03%	3.20%	7.07%
